# Recombinant bovine respiratory syncytial virus with deletion of the SH gene induces increased apoptosis and pro-inflammatory cytokines *in vitro*, and is attenuated and induces protective immunity in calves

**DOI:** 10.1099/vir.0.064931-0

**Published:** 2014-06

**Authors:** Geraldine Taylor, Sara Wyld, Jean-Francois Valarcher, Efrain Guzman, Michelle Thom, Stephanie Widdison, Ursula J. Buchholz

**Affiliations:** 1 Pirbright Institute, Woking, Surrey, GU24 0NF, UK; 2 Laboratory of Infectious Diseases, National Institute of Allergy and Infectious Diseases, Bethesda, MD, USA

## Abstract

Bovine respiratory syncytial virus (BRSV) causes inflammation and obstruction of the small airways, leading to severe respiratory disease in young calves. The virus is closely related to human (H)RSV, a major cause of bronchiolitis and pneumonia in young children. The ability to manipulate the genome of RSV has provided opportunities for the development of stable, live attenuated RSV vaccines. The role of the SH protein in the pathogenesis of BRSV was evaluated *in vitro* and *in vivo* using a recombinant (r)BRSV in which the SH gene had been deleted. Infection of bovine epithelial cells and monocytes with rBRSVΔSH, *in vitro*, resulted in an increase in apoptosis, and higher levels of TNF-α and IL-1β compared with cells infected with parental, wild-type (WT) rBRSV. Although replication of rBRSVΔSH and WT rBRSV, *in vitro*, were similar, the replication of rBRSVΔSH was moderately reduced in the lower, but not the upper, respiratory tract of experimentally infected calves. Despite the greater ability of rBRSVΔSH to induce pro-inflammatory cytokines, *in vitro*, the pulmonary inflammatory response in rBRSVΔSH-infected calves was significantly reduced compared with that in calves inoculated with WT rBRSV, 6 days previously. Virus lacking SH appeared to be as immunogenic and effective in inducing resistance to virulent virus challenge, 6 months later, as the parental rBRSV. These findings suggest that rBRSVΔSH may be an ideal live attenuated virus vaccine candidate, combining safety with a high level of immunogenicity.

## Intoduction

Bovine respiratory syncytial virus (BRSV), which is a major cause of respiratory disease in young calves (Stott & Taylor, 1985), is closely related to human (H)RSV, which is the single most important cause of bronchiolitis and pneumonia in children under 5 years of age (Nair *et al.*, 2010). The high degree of genetic and antigenic similarity between HRSV and BRSV, and the similar epidemiology and pathogenesis of infection, indicate that comparative studies of the immunobiology of these viruses will yield important insights that should benefit both man and cattle. BRSV and HRSV primarily infect ciliated airway epithelial cells and induce a robust inflammatory response in the airways which contributes to the development of bronchiolitis and interstitial pneumonia (Johnson *et al.*, 2007; Viuff *et al.*, 2002; Welliver *et al.*, 2007). BRSV and HRSV belong to the *Pneumovirus* genus, within the family *Paramyxoviridae*, and contain a single-stranded, negative-sense RNA genome encoding 11 proteins: 2 non-structural proteins (NS1 and NS2), nucleocapsid (N) protein, phosphoprotein (P), matrix protein (M), glycoproteins SH, G (attachment) and F (fusion), M2-1 and M2-2 which control transcription and RNA replication, and the RNA polymerase (L).

Despite the economic impact of BRSV, current commercially available inactivated and live, attenuated BRSV vaccines are poorly effective, especially in young calves with maternally derived serum antibodies (MDA), as the duration of protective immunity is short (Meyer *et al.*, 2008). There are also safety concerns associated with the use of inactivated BRSV vaccines, which have the potential to prime for exacerbated disease following subsequent BRSV infection (Schreiber *et al.*, 2000), an observation reminiscent of that seen in babies vaccinated with formalin-inactivated HRSV (Kim *et al.*, 1969). The ability to manipulate the genome of RSVs has allowed analysis of the role of viral proteins in the pathogenesis of disease and has provided opportunities for the development of stable, live attenuated virus vaccines. However, a problem with this approach has been that attenuation is usually based on decreased virus replication, which is associated with reduced immunogenicity. Although an effective HRSV vaccine is not yet available, one promising candidate is a temperature-sensitive mutant virus that also lacks the SH gene, rA2cp248/404/1030/ΔSH. Following intranasal (i.n.) vaccination of 1- to 2-month-old infants, this virus was well tolerated and immunogenic, and protected against a second dose of the same vaccine given 4 to 8 weeks after the first dose (Karron *et al.*, 2005).

The SH protein, which is a short type II integral membrane glycoprotein, is not essential for virus replication in cell culture. However, HRSV lacking the SH gene (rHRSVΔSH) exhibited different patterns of site-specific attenuation in the respiratory tract of mice and chimpanzees. Thus, replication of rHRSVΔSH was attenuated only in the upper respiratory tract of mice (Bukreyev *et al.*, 1997), and only in the lower respiratory tract of chimpanzees (Whitehead *et al.*, 1999). Nevertheless, chimpanzees infected with rHRSVΔSH developed less rhinorrhoea than those infected with WT HRSV. The reasons for differences in the site-specific attenuation of rHRSVΔSH in mice and chimpanzees are not clear. Studies of rHRSVΔSH in cell culture suggest that, similar to other paramyxoviruses, the SH protein inhibits apoptosis and TNF-α signalling (Fuentes *et al.*, 2007; Lin *et al.*, 2003). The HRSV SH protein forms pentameric and hexameric complexes in membranes, altering membrane permeability, and the transmembrane domain of SH has been shown to act as an ion channel in synthetic membranes (Carter *et al.*, 2010; Gan *et al.*, 2012; Perez *et al.*, 1997). Transmembrane pore-forming viral proteins that form ion channels (viroporins), such as influenza virus M2 and encephalomyocarditis virus 2B, play a central role in activation of the NLRP3 inflammasome pathway resulting in secretion of IL-1β and IL-18 (Ichinohe *et al.*, 2010; Ito *et al.*, 2012). There is evidence that HRSV also activates the NLRP3 inflammasome, inducing secretion of IL-1β (Segovia *et al.*, 2012; Triantafilou *et al.*, 2013).

IL-1β and IL-18 play an important role in inflammation by orchestrating the pro-inflammatory response, and are important regulators of innate and adaptive immune responses. These cytokines are produced as cytosolic precursors that require proteolytic cleavage induced by the inflammasome for activation and secretion. BRSV and HRSV induce the production of pro-inflammatory cytokines, including IL-β, *in vivo* and *in vitro* (Bermejo-Martin *et al.*, 2007; Fach *et al.*, 2010; Werling *et al.*, 2002). Activation of the NLRP3/ASC inflammasome by HRSV in mouse bone marrow macrophages is initiated by Toll-like receptor-2 (TLR-2)/Myd88/NF-κB signalling (signal 1), and reactive oxygen species (ROS) and K^+^ ion efflux (signal 2) (Segovia *et al.*, 2012). In contrast, studies in human lung epithelial cells indicated that activation of the NLRP3/ASC inflammasome by HRSV was initiated by TLR-4 (signal 1) and involved the SH protein of RSV (signal 2) (Triantafilou *et al.*, 2013). Thus, rHRSVΔSH failed to induce IL-1β secretion from primary human epithelial lung cells.

Activation of the inflammasome and production of IL-1β is responsible for neutrophil recruitment and inflammation in the lungs of mice infected with influenza virus (Ichinohe *et al.*, 2009; Schmitz *et al.*, 2005; Thomas *et al.*, 2009). The demonstration that rHRSVΔSH failed to induce IL-1β secretion in airway epithelial cells (Triantafilou *et al.*, 2013) may explain, at least in part, the reduced rhinorrhoea seen in chimpanzees infected with rHRSVΔSH (Whitehead *et al.*, 1999). However, activation of the inflammasome plays an important role in orchestrating the innate and adaptive immune response, and mice deficient in components of the inflammasome pathway or which lack IL-18 receptors are more susceptible to influenza virus infection than wild-type mice and have reduced T-cell and antibody responses (Ichinohe *et al.*, 2009; Schmitz *et al.*, 2005). These observations suggest that failure to induce IL-1β by rHRSVΔSH may have a negative impact on priming of a protective immune response, and consequently, deletion of SH would be expected to enhance virus replication in the respiratory tract, rather than attenuate replication as has been observed in mice and chimpanzees.

In order to understand the role of the SH protein in induction of IL-1β and the pathogenesis of RSV, we analysed the effects of deletion of the SH gene on the induction of pro-inflammatory cytokines and apoptosis *in vitro* and on the pathogenesis of BRSV in calves, a natural host of the virus. In addition, the ability of mucosal immunization with rBRSVΔSH to induce a protective immune response was evaluated.

## Results

### Induction of apoptosis by rBRSV lacking the SH gene

**Fig. 1.**
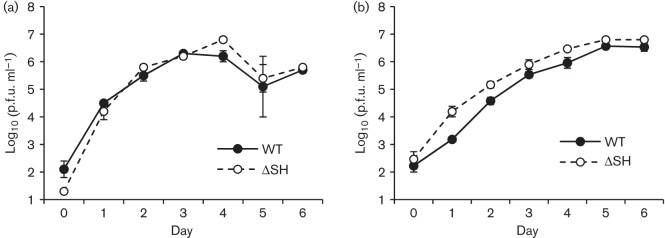
Replication of rBRSV and rBRSVΔSH in (a) calf testes cells and (b) MDBK cells. Triplicate cell monolayers in 6-well plates were infected at an m.o.i. of 0.1. Cells were harvested at daily intervals and stored at −70 °C. Values are the mean log_10_(p.f.u. ml^−1^)±sd of triplicate wells.

**Fig. 2.**
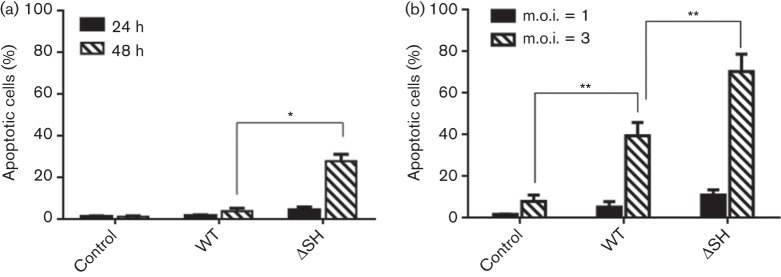
BRSV lacking the SH gene induces apoptosis in MDBK cells and bovine monocytes. (a) MDBK cells were infected with WT rBRSV or rBRSV lacking the SH gene (ΔSH) at an m.o.i. of 3. (b) Bovine monocytes were infected with WT or ΔSH rBRSV at an m.o.i. of 1 or 3. As a control, cells were exposed to mock-infected tissue culture cell lysate. The proportions of apoptotic cells were determined 24 and 48 h p.i. of MDBK cells, or 24 h p.i. of monocytes, using a TUNEL assay and flow cytometry. Results are expressed as the mean percentage apoptotic cells±sd of triplicate samples. *, *P*<0.0001; **, *P*<0.01.

Compared with parental rHRSV, rHRSV lacking SH produces larger plaques and grows to slightly higher titres in some, but not other cell lines (Bukreyev *et al.*, 1997), and induces significantly more apoptosis than parental rHRSV in L929 and A549 cells (Fuentes *et al.*, 2007). Although we found that there was little or no difference either in the plaque size of parental, WT rBRSV and rBRSVΔSH in Vero cells (data not shown), or in the replication of rBRSVΔSH and WT rBRSV in either primary calf testes or MDBK cells (Fig. 1), a cytopathic effect was seen earlier in MDBK cells infected with rBRSVΔSH than in cells infected with WT rBRSV. Infection of MDBK cells or bovine monocytes (CD14^+^ cells) with rBRSVΔSH induced significantly more apoptosis than WT rBRSV, 48 h or 24 h after infection, respectively (*P*<0.0001, *P*<0.01) (Fig. 2a, b). Apoptosis of bovine monocytes 24 h after infection was only seen at an m.o.i. of 3 and there was little or no apoptosis at an m.o.i. of 1 (Fig. 2b).

### Induction of pro-inflammatory cytokines

**Fig. 3.**
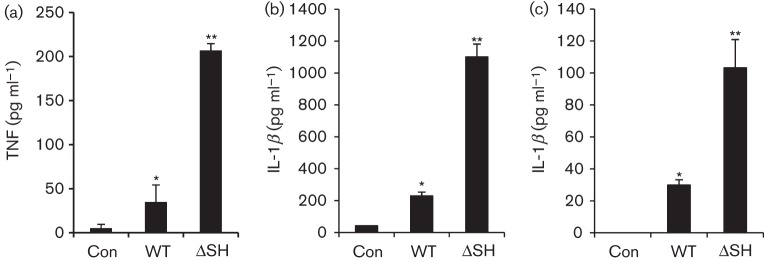
BRSV lacking the SH gene induces secretion of high levels of TNF-α and IL-1β. Bovine monocytes were infected with WT rBRSV or rBRSV lacking the SH gene (ΔSH) at an m.o.i. of 1 (a, b); and MDBK cells were infected with WT or ΔSH rBRSV at an m.o.i. of 3 (c). As controls, cells were exposed to mock-infected tissue culture cell lysate (Con). At 24 h post-infection, levels of TNF-α (a) and IL-1β (b, c) in the supernatant were determined by ELISA. Results are expressed as the mean±sd of triplicate samples. *, WT rBRSV induced significantly higher levels of TNF-α or IL-1β than controls, *P*<0.03; **, rBRSVΔSH induced significantly higher levels of TNF-α or IL-1β than WT rBRSV, *P*<0.0001.

Previous studies have shown that the SH protein of paramyxoviruses plays an important role in inhibiting induction of TNF-α (Fuentes *et al.*, 2007; Lin *et al.*, 2003). We therefore analysed the effect of infection with rBRSVΔSH on the level of TNF-α in the supernatant from bovine monocytes. Although neither WT virus nor rBRSVΔSH replicated in bovine monocytes, cells infected with rBRSVΔSH produced significantly greater amounts of TNF-α than those infected with the parental WT rBRSV (Fig. 3a). Similarly, the level of IL-1β in supernatants from bovine monocytes and MDBK cells infected with rBRSVΔSH was significantly greater than that from cells infected with WT rBRSV (Fig. 3b, c).

### The SH-deleted mutant of BRSV is attenuated in the lower respiratory tract of calves

**Fig. 4.**
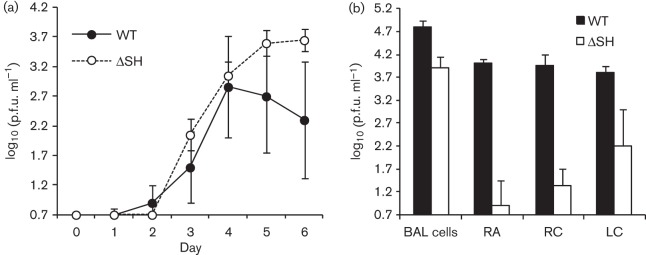
Replication of SH deletion mutant of BRSV in the respiratory tract of calves. Two-to-three-week-old gnotobiotic calves were inoculated i.n. and i.t. with 5×10^6^ p.f.u. WT rBRSV (*n* = 4) or rBRSV lacking the SH gene (ΔSH) (*n* = 3). (a) Nasopharyngeal excretion of virus expressed as the mean log_10_(p.f.u. ml^−1^)±sd. (b) Mean titre of virus [log_10_(p.f.u.)±sd] in BAL cells, or homogenates of samples from the right apical (RA), right cardiac (RC) and left cardiac (LC) lobes of the lung, 6 days after infection.

Since previous studies have shown that there are differences in the site-specific attenuation of rHRSVΔSH in mice and chimpanzees, we compared the replication of WT rBRSV and rBRSVΔSH in the respiratory tract of gnotobiotic calves following i.n. and intratracheal (i.t.) inoculation. Replication of WT and ΔSH rBRSV in the nasopharynx of calves was not significantly different, at least up to 6 days post-infection (p.i.) (Fig. 4a). In contrast, replication of rBRSVΔSH in the lower respiratory tract was attenuated compared with that of WT rBRSV (Fig. 4b). Thus, levels of virus were significantly (*P*<0.01) higher in lung homogenates from calves infected with the WT virus than from those infected with the ΔSH mutant, for samples taken from all lobes. Furthermore, the titre of rBRSVΔSH in bronchiolar lavage (BAL) cells was 10-fold less than that of WT rBRSV, although the difference was not statistically significant.

**Fig. 5.**
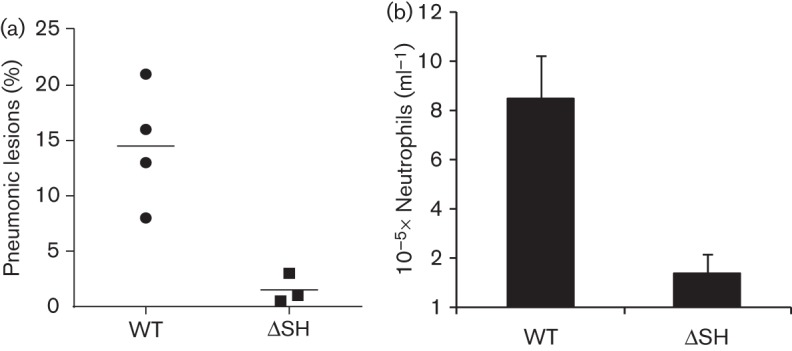
rBRSV lacking the SH protein induces less pulmonary pathology than WT rBRSV. (a) Macroscopic lung lesions in calves infected 6 days previously with 5×10^6^ p.f.u. rBRSV lacking the SH gene (ΔSH) were significantly reduced compared with those in calves infected with WT rBRSV (*P*<0.02). (b) The number of neutrophils in BAL (mean±sd), 6 days after infection. The number of neutrophils in BAL from calves infected with rBRSVΔSH was significantly reduced compared with that in calves infected with WT virus (*P*<0.05).

**Fig. 6.**
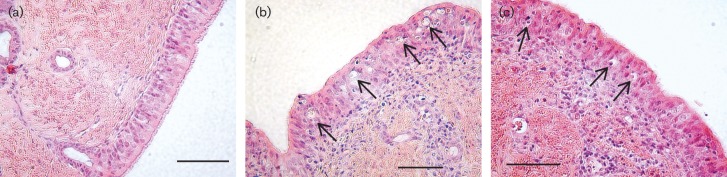
Histopathological changes in tracheal epithelium from BRSV-infected gnotobiotic calves. Vacuoles containing cell debris (arrows) were detected in tracheal epithelium from calves inoculated i.n. and i.t., 6 days previously, with WT rBRSV (b) or rBRSVΔSH (c), but not in the epithelium from calves inoculated with control Vero cell lysate (a). Bar represents 100 µM.

None of the calves developed clinical signs of respiratory disease. However, in contrast to calves infected with WT rBRSV, calves infected with rBRSVΔSH developed little or no macroscopic pneumonia (Fig. 5a). Furthermore, the total number of cells in BAL from calves infected with rBRSVΔSH was twofold less than that from calves infected with WT rBRSV, and the numbers of neutrophils in BAL were significantly reduced (Fig. 5b). Microscopic lung lesions in calves infected with WT rBRSV were similar to those described previously (Thomas *et al.*, 1984) and were characterized by a proliferative and exudative bronchiolitis, a peribronchiolar accumulation of lymphocytes and alveolitis. Microscopic lung lesions in calves infected with rBRSVΔSH were similar but much less extensive. Numerous apoptotic cells can be seen in vacuoles in bronchial epithelial cells of BRSV-infected calves (Viuff *et al.*, 2002). Since rBRSVΔSH infection increased apoptosis, *in vitro*, we analysed sections of trachea and lung from calves infected with WT or ΔSH rBRS viruses for vacuoles containing cell debris. Such vacuoles were observed in bronchial and tracheal epithelial cells from calves infected with either WT rBRSV or rBRSVΔSH, but not in airway epithelium from mock-infected calves (Fig. 6). Although microscopic changes were not very extensive in calves infected with rBRSΔSH, there did not appear to be any major differences in the numbers of vacuoles containing cell debris in areas of lung sections showing inflammation when compared with sections from calves infected with WT rBRSV.

### Mucosal immunization with rBRSV lacking SH protects against challenge with virulent BRSV

**Fig. 7.**
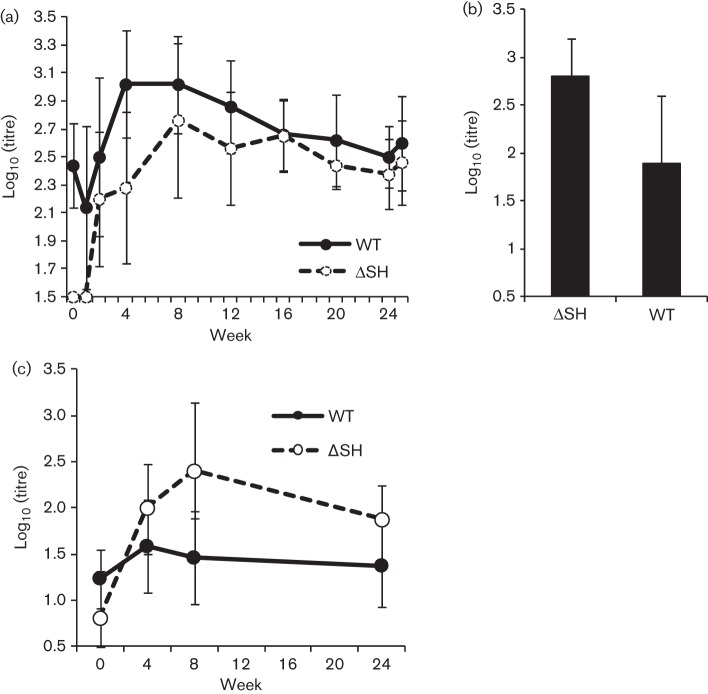
BRSV-specific antibody responses induced by mucosal vaccination of calves with rBRSV. Calves were inoculated i.n. and i.t. with 5×10^6^ p.f.u. WT rBRSV (*n* = 5) or rBRSV lacking the SH gene (ΔSH) (*n* = 4). Calves were challenged 24 weeks after vaccination with 1×10^4^ p.f.u. of the Snook strain of BRSV in BAL. BRSV-specific IgG antibody responses in sera (a) and BRSV-specific IgA antibody in nasal secretions, 2 weeks after vaccination (b) were determined by ELISA. (c) BRSV-specific serum neutralizing antibody responses were determined by a plaque reduction assay. Results are expressed as the geometric mean titre (log_10_) ±sd.

In order to determine the ability of mucosal immunization with rBRSVΔSH to induce a protective immune response, groups of four or five colostrum-restricted calves that had been inoculated i.n. and i.t. with WT rBRSV, rBRSVΔSH or control Vero cell lysate, 6 months previously, were challenged with the Snook strain of BRSV. As seen previously in gnotobiotic calves, there was no difference in the replication of WT and ΔSH rBRS viruses in the nasopharynx, and virus was cleared by day 8 in both groups of infected animals. Furthermore, none of the calves developed clinical signs of respiratory disease. Although calves infected with WT rBRSV had higher levels of maternally derived BRSV-specific serum antibodies at the time of mucosal immunization than calves infected with rBRSVΔSH, the serum IgG antibody response, as determined by ELISA, and the neutralizing antibody response induced by rBRSV and rBRSVΔSH were not statistically significantly different (Fig. 7a, c). Infection with WT or ΔSH rBRSV also induced BRSV-specific IgA in nasal secretions. Mucosal IgA reached a peak at 2 weeks after vaccination in both groups of calves and the titres were not significantly different (Fig. 7b). After a peak between 4 and 8 weeks after immunization, levels of BRSV-specific serum IgG and neutralizing antibodies declined slowly over the 6 months. In contrast, mock-infected control calves did not develop a BRSV-specific serum or mucosal antibody response (results not shown).

**Fig. 8.**
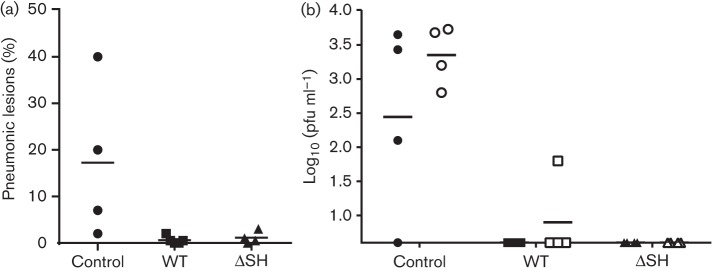
Recombinant BRSV lacking SH protects against challenge with virulent BRSV. Calves were inoculated i.n. and i.t. with 5×10^6^ p.f.u. WT rBRSV (*n* = 5) or rBRSV lacking the SH gene (ΔSH) (*n* = 4). As controls, calves were inoculated i.n. and i.t with control tissue culture cell lysate. Calves were challenged 24 weeks after vaccination with 1×10^4^ p.f.u. of the Snook strain of BRSV in BAL and killed 6 days after challenge. (a) Percentage of the lung showing macroscopic lung lesions, 6 days after challenge. (b) Peak virus titres in the nasopharynx (solid symbols) and titre of virus in BAL cells, 6 days after challenge (open symbols).

Following challenge with virulent BRSV, clinical signs of respiratory disease were seen 5 to 6 days after challenge in two out of four control calves, one out of four calves immunized with rBRSVΔSH and none of the calves immunized with WT rBRSV. At post-mortem examination, 6 days after BRSV challenge, macroscopic lung lesions of >5 % of the lung were observed in three out of four control calves (Fig. 8a). In contrast, there was little or no pneumonic consolidation in calves immunized with either WT rBRSV or rBRSVΔSH (Fig. 8a). Following challenge, virus was not isolated from the nasopharynx or lung tissue of any of the immunized calves but was isolated from the nasopharynx (Fig. 8b) and lung tissue of three of the four control calves (results not shown). Similarly, virus was not isolated, or only detected in low titres, in BAL cells from calves immunized with rBRSVΔSH or WT rBRSV, but was isolated in high titres from control calves (Fig. 8b).

## Discussion

In this study, we demonstrated that although BRSV lacking the SH gene replicated as efficiently as WT rBRSV in tissue culture cells, the mutant virus exhibited site-specific attenuation in the bovine respiratory tract, and was as effective as WT rBRSV in inducing resistance to challenge with virulent BRSV, 6 months after vaccination. Although previous studies have shown that the final titre of rBRSVΔSH in MDBK cells was slightly reduced when compared with that of parental rBRSV (Karger *et al.*, 2001), we could not detect any differences in the replication of rBRSVΔSH and parental WT rBRSV in either calf testes or MDBK cells. Studies with rHRSV lacking the SH gene (D46/6368) showed that the mutant virus formed larger plaques on Hep-2 cells, and replicated to higher titres than parental rHRSV in some, but not all, cell lines tested (Bukreyev *et al.*, 1997). Therefore, differences in the replication of rBRSVΔSH, *in vitro*, may be related to the use of different cell lines, or to differences in cell lines maintained in different laboratories. Taken together, studies on rHRSV and rBRSV lacking the SH protein demonstrate that SH is not essential for virus replication, *in vitro*. In contrast, the replication of rBRSVΔSH was attenuated in the lungs of calves. Thus, the site-specific attenuation of rBRSVΔSH in calves resembled that of rHRSVΔSH in chimpanzees (Whitehead *et al.*, 1999).

Although the parental WT rBRSV did not induce clinical signs of disease, it induced an extensive pulmonary inflammatory response and gross pneumonic lesions. Attempts to induce clinical signs of respiratory disease in calves experimentally infected with BRSV have yielded inconsistent results. However, non- or low-cell-culture-passaged virus can induce disease similar to that seen in naturally occurring outbreaks (van der Poel *et al.*, 1996). In the field, other factors such as secondary infections, adverse environmental temperatures or housing may contribute to the severity of disease. Nevertheless, the marked reduction in lung pathology seen in calves infected with rBRSVΔSH compared with that induced by WT rBRSV suggests that SH plays a role in RSV pathogenesis.

HRSV and BRSV do not usually induce apoptosis until the late stages of infection (Cristina *et al.*, 2001; Kotelkin *et al.*, 2003). However, rBRSVΔSH resembled rHRSVΔSH in inducing significantly more apoptosis than WT virus, *in vitro* (Fuentes *et al.*, 2007). Studies with rPIV5ΔSH have suggested that the SH protein inhibits TNF-α-induced apoptosis (Lin *et al.*, 2003). Like rPIV5ΔSH, we found that rBRSVΔSH-infected bovine monocytes produced significantly greater amounts of TNF-α than cells infected with WT rBRSV. Therefore, the greater level of apoptosis seen in rBRSVΔSH-infected cells compared with cells infected with WT rBRSV may be mediated by the higher levels of TNF-α induced in rBRSVΔSH-infected cells. However, apoptosis of bovine monocytes, 24 h after infection with rBRSVΔSH at an m.o.i. of 1, was low at a time when high levels of TNF-α were present in the supernatant.

Apoptosis is an important early host defence that limits virus replication and spread, and many viruses have developed strategies to inhibit apoptosis, thereby prolonging their ability to replicate. An early onset of apoptosis following virus infection may help to control virus replication, as seen for rPIV5ΔSH, which induced increased apoptosis, *in vitro*, and was attenuated *in vivo* (He *et al.*, 2001). The observation that a large number of apoptotic cells containing BRSV antigen can be seen in vacuoles in the bronchial epithelium of calves infected 6 days previously with BRSV has led to the suggestion that apoptosis is an important pathway of virus clearance in BRSV-infected calves (Viuff *et al.*, 2002). Therefore, an accelerated or increased induction of apoptosis by rBRSVΔSH may explain its restricted replication in the lungs. However, this does not explain the similar level of replication and rate of clearance of rBRSVΔSH and WT rBRSV seen in the nasopharynx. It is possible that the higher pro-apoptotic potential of rBRSVΔSH could predispose the airways to secondary bacterial infections. However, we found no evidence for this, and, furthermore, we could not detect any major differences in the number of vacuoles containing cell debris in ciliated airway epithelium in calves infected 6 days previously with rBRSVΔSH or WT rBRSV. An alternative explanation for the site-specific attenuation of rBRSVΔSH may be related to induction of TNF-α by monocytes and macrophages, which are more prevalent in the lower respiratory tract than in the nasopharynx. Although TNF-α is a major mediator of RSV-associated illness in BALB/c mice, it is important in clearance of virus-infected cells during the early stages of infection (Rutigliano & Graham, 2004).

The observation that rBRSVΔSH induced significantly higher levels of IL-1β than WT rBRSV in MDBK cells and bovine monocytes contrasts with that of Triantafilou *et al.* (2013), who found that rHRSVΔSH did not induce secretion of IL-1β in primary human lung epithelial cells. The reasons for this discrepancy are not clear. One possibility is that, since there is only 38 % amino acid identity between the SH proteins of BRSV and HRSV, they may have different functions. However, the close relationship between HRSV and BRSV makes this unlikely. The predicted hydropathy profiles of BRSV and HRSV SH proteins are similar (Samal & Zamora, 1991) and, of the amino acids (His22, and either His51 or Trp15) that have been implicated in HRSV SH ion channel activity (Carter *et al.*, 2010; Gan *et al.*, 2012), Trp15 and His51 are conserved in the BRSV SH protein. Since activation of the inflammasome by HRSV is mediated by different signals in different cells (Segovia *et al.*, 2012; Triantafilou *et al.*, 2013), it is possible that differences in induction of IL-1β by rHRSVΔSH and rBRSVΔSH may be related to the type of cells that were infected. However, rBRSVΔSH induced higher levels of IL-1β than WT rBRSV in both bovine epithelial (MDBK) cells and bovine monocytes. Alternatively, differences in the response to infection with ΔSH RS viruses may be related to differences in their construction resulting in differing levels of read-through transcripts, and consequently, the level of expression of downstream viral genes. Thus, differences in the intergenic regions, or expression of an additional gene such as GFP, as in the rHRSVΔSH used in studies by Triantafilou *et al.* (2013), may have affected the gradient of transcription and the level of expression of, for example, the G and F proteins. The G protein of HRSV inhibits IL-6 and IL-1β production in human monocytes (Polack *et al.*, 2005). Therefore, differences in the level of G protein produced by ΔSH viruses may influence induction of IL-1β. There are high levels of read-through mRNAs of the M gene and the respective downstream genes in rHRSV (D46/6368) (Bukreyev *et al.*, 1997) and rBRSVΔSH (Karger *et al.*, 2001). However, this does not appear to translate to corresponding higher levels of downstream proteins in cells infected with rHRSV (D46/6368). Other studies have shown that TNF-α can induce caspase-1 activation and IL-1β secretion (Dinarello *et al.*, 1986; Franchi *et al.*, 2009). Therefore, it is possible that increased secretion of IL-1β by cells infected with rBRSVΔSH was mediated by TNF-α.

Studies in mice infected with influenza virus have demonstrated that IL-1β is responsible for neutrophil recruitment and inflammation in the lungs (Ichinohe *et al.*, 2009; Schmitz *et al.*, 2005; Thomas *et al.*, 2009). Increased secretion of IL-1β by bovine monocytes infected with rBRSVΔSH might be expected to result in increased inflammation in the lungs of calves infected with this virus. However, the pulmonary inflammatory response in calves infected with rBRSVΔSH was reduced in comparison with that seen in calves infected with WT rBRSV 6 days previously. Early induction of IL-1β and TNF-α by rBRSVΔSH in the lungs may have contributed to rapid control of virus replication and, therefore, less inflammation, 6 days after infection. Further studies to compare innate immune responses in the lungs of calves infected with rBRSVΔSH or WT rBRSV are required to understand the mechanisms responsible for the reduced virus replication and reduced pulmonary inflammatory response in calves infected with rBRSVΔSH.

Despite the attenuation of rBRSVΔSH in the lower respiratory tract of calves, BRSV-specific antibody responses induced by the mutant virus were similar to those induced by WT rBRSV. Furthermore, infection with rBRSVΔSH was as effective as that with WT rBRSV in protecting against BRSV challenge 6 months later. The observation that attenuation of rBRSVΔSH did not appear to be associated with reduced immunogenicity may be related to increased activation of components of the innate immune response, such as apoptosis and IL-1β. Apoptosis enhances antigen presentation, and apoptotic cells or bodies are major sources for antigen cross-presentation. An increase in apoptosis by a recombinant rabies virus overexpressing cytochrome *c* resulted in attenuation of pathogenicity and enhanced immunity (Pulmanausahakul *et al.*, 2001). Similarly, the enhanced immune response and greater vaccine efficacy against influenza virus induced in mice by rPIV5ΔSH expressing influenza haemagglutinin was associated with increased apoptosis, *in vitro*, and attenuation of virus replication, *in vivo* (Li *et al.*, 2013). Activation of the inflammasome and induction of IL-1β and IL-18 secretion also appear to be important for induction of adaptive immunity. Thus, priming of influenza virus-specific T cells, and levels of nasal IgA were significantly reduced in mice deficient in ASC, caspase-1 or IL-1R (Ichinohe *et al.*, 2009); and deletion of the gene encoding the viral IL-1β receptor in modified vaccinia virus Ankara enhanced the induction of memory CD8 T cells (Staib *et al.*, 2005). However, other studies have failed to demonstrate a role for caspase-1 in induction of the adaptive immune response to influenza virus (Thomas *et al.*, 2009).

In conclusion, rBRSVΔSH showed site-specific attenuation in the respiratory tract of calves, and was as immunogenic and effective as WT rBRSV in inducing resistance to challenge with virulent BRSV. These properties may be related to an increased induction of components of the innate immune response, such as apoptosis and IL-1β, in rBRSVΔSH-infected cells. In contrast to a biologically derived vaccine candidate attenuated by point mutations, rBRSVΔSH containing the deletion of an entire gene should be highly refractory to reversion to virulence. Furthermore, the probability of regaining the deleted gene by recombination is likely to be a very rare event (Spann *et al.*, 2003). As the fusion glycoprotein, which is the major protective antigen, is highly conserved between BRSV isolates (Valarcher *et al.*, 2000), immune responses induced by SH-deleted BRSV are likely to be broadly protective in the field. Therefore, rBRSVΔSH appears to be an ideal vaccine candidate, combining safety with a high level of immunogenicity.

## Methods

### 

#### Viruses and cells.

WT recombinant (r)BRSV and virus lacking the SH gene (ΔSH) were derived from full-length cDNA of BRSV strain A51908, variant Atue51908 (GenBank accession no. AF092942) (Karger *et al.*, 2001). Stocks of rBRSV were prepared in Vero cell monolayers, infected at an m.o.i. between 0.1 and 0.5, in Dulbecco`s modified Eagle’s medium (DMEM; Gibco-BRL) containing 2 % heat-inactivated fetal calf serum (FCS). All recombinant virus stocks were free from contamination with bovine viral diarrhea virus (BVDV) and mycoplasmas. Virulent BRSV consisted of BAL prepared from a gnotobiotic calf, inoculated 6 days previously with the Snook strain of BRSV, which is closely related to BRSV Atue51908 (Valarcher *et al.*, 2003). The BAL was free from other viruses, mycoplasmas and bacteria as assessed by inoculation of tissue culture cells, or mycoplasmal or bacterial media. Replication of rBRSV and rBRSVΔSH in primary calf testes cells and MDBK cells, infected at an m.o.i. of 0.1 and incubated at 37 °C or 6 days, was determined as described previously (Valarcher *et al.*, 2003). Virus titres were determined by plaque assay on fetal calf kidney (FCK) or Vero cell monolayers in 35 mm Petri dishes (Thomas *et al.*, 1982).

#### Calves and experimental design.

Gnotobiotic, BRSV-seronegative calves were derived, reared and maintained individually in plastic isolators as described previously (Dennis *et al.*, 1976). To evaluate the virulence of rBRSV, groups of three or four gnotobiotic calves were infected at 2 to 3 weeks of age with approximately 10^6^ p.f.u. virus in a volume of 10 ml administered i.n. and 10 ml i.t. A clinical examination was performed twice a day following virus infection, and nasopharyngeal swabs were obtained daily to monitor virus excretion from the nasopharynx. Calves were killed 6 days after infection, by intravenous injection of sodium pentobarbital. At post-mortem examination, macroscopic lung lesions were recorded on a standard lung diagram, and the extent of pneumonic consolidation was expressed as a percentage of the lung area. BAL was collected by irrigating the lungs from each calf with 300 ml PBS (Taylor *et al.*, 1995). Cytocentrifuge preparations of BAL cells were fixed in neutral buffered formalin and stained with haematoxylin and eosin. Differential counts of 300 to 350 cells per slide were made using oil immersion. Cells prepared from 100 ml BAL by centrifugation at 1200 ***g*** for 15 min at 4 °C were resuspended in 5 ml of lung buffer for analysis of virus titre (Taylor *et al.*, 1995). Three pieces of pneumonic lung taken from different lobes were homogenized in lung buffer to give a 20 % (w/v) suspension. Titres of rBRSV in nasopharyngeal swabs, BAL cells and lung homogenates were determined by plaque assay on Vero cells.

The ability of rBRSVΔSH to induce protective immunity was determined in three groups of colostrum-restricted calves, produced by collecting them at birth and artificially feeding them with 500 ml of colostrum that contained low levels of BRSV-specific antibodies. Calves were inoculated i.n. and i.t. at 1 to 4 weeks of age, with 5×10^6^ p.f.u. WT rBRSV, rBRSVΔSH or a lysate of non-infected Vero cells. Nasopharyngeal swabs were obtained daily for 10 days to monitor virus excretion from the nasopharynx. Blood was obtained at intervals for analysis of BRSV-specific antibodies. Six months after vaccination, calves were challenged i.n. and i.t. with 1×10^4^ p.f.u. of the virulent Snook strain of BRSV in BAL fluid. Following challenge, nasopharyngeal swabs were obtained daily, and calves were killed 6 days after challenge to determine the extent of gross pneumonic lesions and the titre of virus in BAL cells.

All calf experiments were performed under the regulations of the Home Office Scientific Procedures Act (1986) of the UK. The studies have been approved by the Pirbright Institute Animal Welfare & Ethical Review Body.

#### Serology.

BRSV-specific antibodies in sera were analysed by ELISA using a lysate of BRSV (Snook)-infected FCK cells as antigen and mock-infected FCK cells as control antigen (Taylor *et al.*, 1995). Neutralizing antibodies were determined by a plaque reduction assay on Vero cells using the Snook strain of BRSV and heat-inactivated (56 °C for 30 min) sera (Kennedy *et al.*, 1988).

#### Histology.

A tracheal ring taken from approximately 5 cm below the larynx and pieces of lung from each of three different lobes were fixed in 10 % neutral buffered formalin and embedded in paraffin wax, and sections were stained with haematoxylin and eosin.

#### Induction of pro-inflammatory cytokines and apoptosis, *in vitro*.

Bovine monocytes were prepared from heparinized venous blood by centrifugation at 1200 ***g*** over Histopaque 1086 (Sigma). The peripheral blood mononuclear cells were washed three times with PBS and CD14^+^ cells were purified by magnetic antibody cell sorting using anti-human CD14^+^ microbeads (Miltenyi Biotec) (Sopp & Howard, 1997), following the manufacturer’s instructions. CD14^+^ cells in RPMI medium with 3 % FCS, ampicillin (0.1 µg ml^−1^) and 5×10^−5^ M 2-mercaptoethanol and MDBK cells in Eagle’s MEM containing 2 % FCS, 1 % non-essential amino acids, 100 U penicillin ml^−1^, and 100 µg streptomycin ml^−1^ were infected with WT BRSV or BRSVΔSH at an m.o.i. of 1 or 3. Virus was adsorbed for 1.5 h at 37 °C in 5 % CO_2_/air. After adsorption, the cells were washed twice with PBS, fresh medium was added and cells were incubated at 37 °C in 5 % CO_2_/air. As a control, cells were treated with mock-infected Vero cell lysate for 1.5 h. At 24 h post-infection, supernatants were collected and levels of IL-1β were analysed using a bovine IL-1β ELISA kit (Pierce Protein Biology Products), and levels of TNF-α were analysed by ELISA as described previously (Kwong *et al.*, 2010). At 24 h post-infection, bovine monocytes were removed from tissue culture wells using cell-dissociation fluid solution (Sigma-Aldrich), and MDBK cells were removed at 24 h and 48 h p.i. by treatment with trypsin and EDTA. Terminal deoxynucleotidyltransferase dUTP nick end labelling (TUNEL) was performed using the APO-BRDU kit (AbD Serotec), according to the manufacturer’s instructions, to detect apoptotic cells by dual colour flow cytometry.

#### Statistics.

Data were analysed for statistical significance using either ANOVA or a two-sample *t*-test. *P* values less than 0.05 were considered statistically significant. Viral levels in lung homogenates of calves were analysed using a linear mixed model with log_10_(p.f.u. g^−1^) as the response variable, virus (WT or ΔSH) and lobe (RA, RC or LC) as fixed effects and calf as a random effect. Model selection proceeded by stepwise deletion of non-significant terms (as judged by the Akaike information criterion), starting from a model including virus, lobe and an interaction between them. Once a final model had been constructed, differences between factors were explored using Tukey’s honest significant differences (which corrects for multiple testing). All analyses were implemented in R (R Core Team 2013) (http://www.R-project.org/).
